# Iatrogenic Pancreatitis Post‐Cholecystectomy—A Surgical Paradox: When Cholecystectomy Leads to Pancreatitis, a Case Report

**DOI:** 10.1002/ccr3.71087

**Published:** 2025-10-15

**Authors:** Fazeela Bibi, Janmejay Kumar Singh, Amina Asad, Qurat‐Ul‐Ain Akram, Aiman Sadiq, Babar fayaz, Bilal Aslam, Vohra Maham Hassan, Umama Alam, Muhammad Abdullah Ali, Khalil Elabdi, Said Hamid Sadat

**Affiliations:** ^1^ Jinnah Medical and Dental College Karachi Sindh Pakistan; ^2^ Teerthankar Mahaveer Medical College Moradabad Uttar Pradesh India; ^3^ Nowshera Medical College Nowshera Khyber Pakhtunkhwa Pakistan; ^4^ Dow University of Health Sciences Karachi Pakistan; ^5^ University of Lahore Lahore Pakistan; ^6^ Khyber Medical College Peshawar Pakistan; ^7^ Faculty of Medicine and Pharmacy of Rabat Mohammed V University Rabat Morocco; ^8^ Kabul University of Medical Sciences Abu Ali Ibn Sina Kabul Afghanistan

**Keywords:** cholelithiasis, endoscopic retrograde cholangiopancreatography (ERCP), iatrogenic pancreatitis, idiopathic acute pancreatitis, laparoscopic/open cholecystectomy, pancreatic injury

## Abstract

Iatrogenic pancreatitis is a rare but serious complication of laparoscopic cholecystectomy. Prompt recognition and aggressive management are crucial in young patients, as in this 23‐year‐old case. Early intervention can prevent severe consequences, emphasizing the importance of vigilant post‐operative monitoring and timely intervention.

## 1 Introduction

Acute Pancreatitis is a widespread condition, considered by health care professionals, that impacts the pancreas and is regarded as the most intricate disease of the gastrointestinal tract. Diagnosing and treating diseases of this glandular organ is critical for maintaining the well‐being of the digestive and endocrine systems, impacting the overall health of the body [1]. The cause of acute pancreatitis (AP) should be determined upon admission and is achieved through a comprehensive medical history, physical exam, lab tests, and imaging. Additionally, assessing risk factors and the patient's response to initial treatment helps forecast the AP outcome [2]. This potentially life‐threatening condition is typically triggered by factors such as alcohol use, gallstones, acute on chronic pancreatitis, or idiopathic causes. It can also occur as a complication following surgeries, particularly those involving the pancreas, including hepatobiliary, gastric surgeries, splenorenal shunts, splenectomies, and certain cardiac procedures [3, 4, 5, 6].

Research indicates that microlithiasis and sludge could be responsible for a large proportion of idiopathic acute pancreatitis (IAP). These small stones and sludge, particularly when located in the common bile duct, are often challenging to detect with transabdominal ultrasound. Consequently, patients initially diagnosed with IAP may actually have biliary pancreatitis. To lower the risk of recurrent acute pancreatitis, performing Laparoscopic/Open Cholecystectomy during the same hospital admission is recommended for cases of mild biliary pancreatitis [7]. Endoscopic retrograde cholangiopancreatography (ERCP) is associated with a 2%–10% risk of post‐ERCP pancreatitis (PEP), which can increase to 30%–50% in high‐risk individuals. In up to 5% of cases, PEP may become severe, leading to potentially fatal complications such as multi‐organ failure, acute peripancreatic fluid collections, and, in rare instances, death, which occurs in about 1% of cases [8]. We are reporting the case after Laparoscopic cholecystectomy pancreatitis, which is a very rare entity. Acute pancreatitis essentially requires the presence of at least two of the three mentioned criteria: (A) abdominal pain (more on epigastric region) characteristic of the condition, (B) three‐fold elevation of serum amylase and/or lipase, and (C) typical results from abdominal radiological studies [9]. Serum pancreatic enzymes are considered the most reliable method for diagnosing acute pancreatitis [10].

## 2 Case History and Examination

A 23‐year‐old patient presented to the emergency department of the trauma center, Civil Hospital Karachi, with complaints of fever for 2 days and pain in the epigastric region and at the post‐surgical site for 3 days, following a laparoscopic cholecystectomy performed 5 days prior. The pain started in the epigastric region, radiating to the tip of the shoulder and at the surgical site. The pain was sudden and sharp, aggravated by eating, and relieved with analgesics for a few hours. However, since the last day, the pain had become more severe and was unrelieved by oral analgesics. The pain was accompanied by vomiting, which was non‐projectile, with two episodes per day containing food particles and no associated bleeding. Fever was sudden in onset, intermittent, and undocumented, associated with chills and rigor. The patient had undergone a laparoscopic cholecystectomy for chronic cholecystitis secondary to cholelithiasis 5 days prior to presentation and had no significant past medical history. At the time of presentation, the patient reported normal bowel and bladder movements and appetite.

On examination, the abdomen was soft, with tenderness at the epigastric and infraumbilical surgical site, a tympanic sound on percussion, and normal bowel sounds were noted. All other systemic examinations were unremarkable. Except the patient had mild yellowish discoloration of the sclera. On admission, vitals were: temperature of 101°F, blood pressure of 97/63 mmHg, heart rate 100 bpm, breathing rate of 20 bpm, and saturation (SpO_2_) 96% on room air. The patient weighed 56 kg and was alert and oriented to time, place, and person. Glasgow Coma Scale (GCS) revealed a score of 15/15.

## 3 Diagnosis and Investigations

**FIGURE 1:**
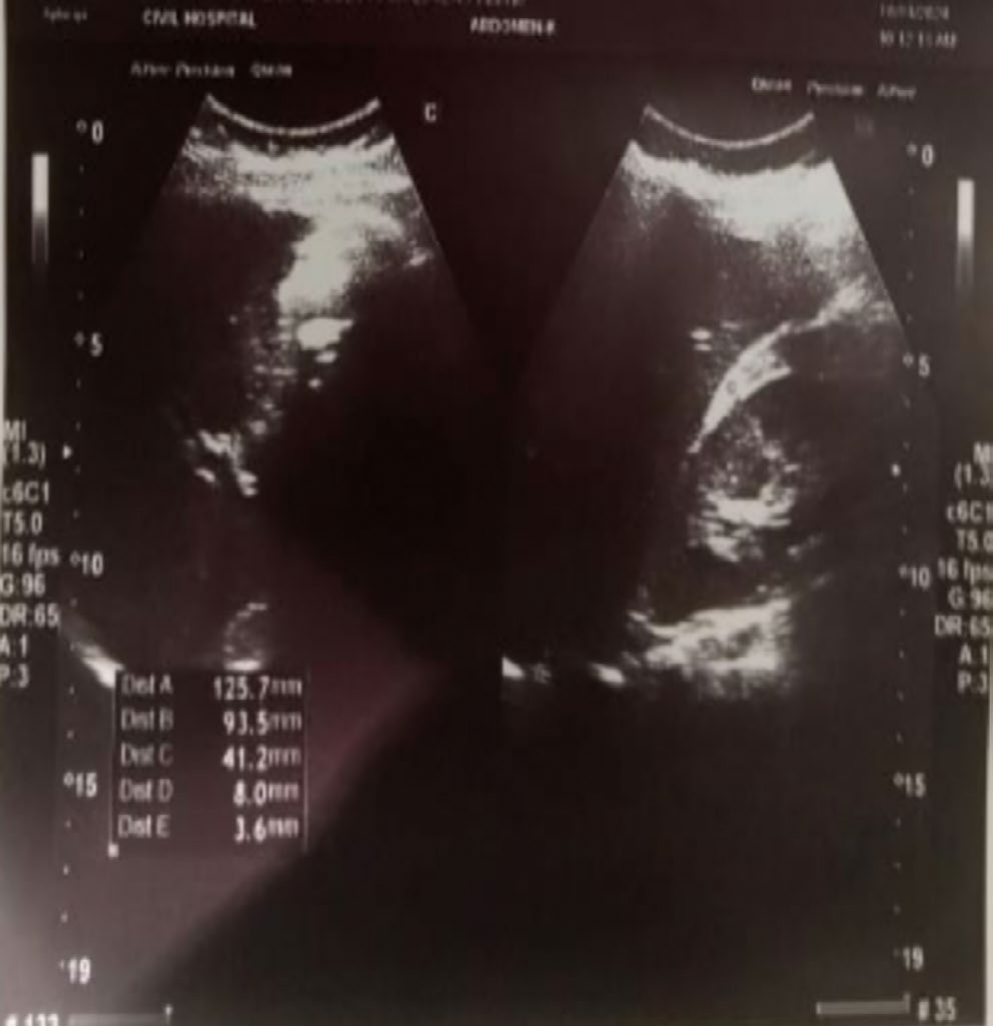
Abdominal ultrasound suggested no dilatation of duct with normal diameter of common bile duct (CBD) of 0.3 cm.

The clinical presentation raised suspicion of iatrogenic acute pancreatitis as a complication of the cholecystectomy. On blood testing, serum amylase (1758 U/L) and lipase (2341 U/L) levels were elevated, respectively. Serum LDH was also elevated at 365 U/L, and a raised total leukocyte count of 16,400/μL was noted. A total bilirubin of 2.6 mg/dL was seen, with direct bilirubin elevated to 1.9 mg/dL. Patients also had raised ALAT of 129, ALP of 210 U/L, and CRP of 130 mg/L at 48 h. No significant findings appeared on urine analysis, and negative results were found in initial blood culture and sensitivity at 48 h. Abdominal ultrasound suggested no dilatation of the duct, with a normal diameter of the common bile duct (CBD) of 0.3 cm (Figure 1). Ranson's score on admission was two (elevated WBC and LDH). On the basis of history, examination, and investigation, iatrogenic acute pancreatitis post‐cholecystectomy was considered to be the final diagnosis.

## 4 Treatment and Follow‐Up

The patient was treated for suspected acute pancreatitis with intravenous (IV) fluids (0.5% dextrose saline), IV analgesics (Acetaminophen and Ketorolac) for pain, IV Acetaminophen for fever, IV pantoprazole, and IV antibiotic (Meropenem 1 g thrice a day). The patient's condition stabilized significantly within the next 48 h, with WBC returning to reference range and a 48‐h Ranson's score of 0. Congruent with the patient's rapid clinical stabilization, the initial nil per os (NPO) status was discontinued after 48 h. Upon the resolution of severe epigastric pain and emesis, oral refeeding was judiciously commenced, beginning with a clear liquid diet. This regimen was subsequently advanced to a low‐fat, solid‐food diet as tolerated by the patient, a step that proceeded without any recurrence of symptoms, thereby facilitating her transfer to the general ward for continued observation. On the third day of admission, the patient was transferred to the general ward and kept under observation until the sixth day. During this time, the patient remained stable, with occasional mild tachycardia that resolved spontaneously. On the sixth day, the patient was discharged with the following oral medications: Cefixime (500 mg) once daily, Omeprazole (40 mg) once daily, Drotaverine (40 mg) twice daily, and Tramadol (50 mg) thrice daily. The patient was advised to avoid spicy and fatty foods and to return for a follow‐up after 1 week. At one‐week follow‐up, the patient was doing well and reported no complaints.

## 5 Discussion

Laparoscopic cholecystectomy (LC) is a widely utilized surgical procedure across the globe, experiencing a significant rise in prevalence over the past decade [11]. Research indicates that approximately 60% of these procedures are performed in women, with a mean age of 40 years and a standard deviation of plus or minus 10 years [12].

Careful surgical technique is essential to prevent post‐cholecystectomy pancreatitis, including thorough dissection around the cystic duct and common bile duct to avoid injury or thermal damage to the pancreatic duct or ampulla. The critical view of safety must be achieved to clearly identify and preserve important biliary and pancreatic anatomy [10, 13]. Excessive traction on the bile duct or pancreas should be avoided to prevent edema or trauma that could trigger pancreatitis [4]. The routine or selective use of intraoperative cholangiography helps identify bile duct stones and anatomical variants, reducing the risk of injury [12]. Additionally, careful preoperative or intraoperative management of common bile duct stones through ERCP or laparoscopic exploration decreases the likelihood of pancreatitis caused by retained stones [7]. Lastly, minimizing manipulation of the ampulla of Vater during surgery prevents inflammation and edema of the pancreatic duct [8].

While this procedure is generally safe, it is important to acknowledge the potential for complications. The most frequently reported complications associated with LC include bile duct injury, bile leaks, bleeding, and bowel injury. Postoperative acute pancreatitis (AP) may occur due to retained stones or other surgical complications, with an estimated incidence between 0.1% and 0.34% [14]. Furthermore, a study conducted in Spain indicated that 7% of all readmissions within 90 days following cholecystectomy were associated with acute pancreatitis [15].

Acute postoperative pancreatitis is a rare complication that can arise following laparoscopic cholecystectomy. In most cases, this condition is effectively managed with conservative treatment without any surgical intervention, except for an active obstruction at the lower common bile duct. During the early postoperative period, acute pancreatitis may be attributed to the passage of a missed gallstone or biliary sludge through the ampulla of Vater. Notably, even biliary microliths can precipitate severe pancreatitis. These microliths have the potential to pass through the common bile duct and traverse the sphincter of Oddi, resulting in a transient obstruction that typically resolves spontaneously. This occurrence is often associated with hyperbilirubinemia, which is generally obstructive. Historical studies have highlighted that small gallstones, which may not be detected by conventional cholecystography techniques, are implicated in up to 75% of idiopathic pancreatitis cases [16].

Acute pancreatitis (AP) can arise from various causes, including alcoholism, certain medications, cystic fibrosis, hypercalcemia, hypertriglyceridemia, and trauma. After excluding these, patients who have recently undergone cholecystectomy should be considered a relevant risk factor. In fact, gallstone pancreatitis, although relatively rare, is a notable risk associated with laparoscopic cholecystectomy (LC), particularly as the likelihood of gallstones entering the biliary tract increases with the number of stones. The tortuous anatomy of the cystic duct can facilitate this passage. While laparoscopic procedures typically have lower complication rates, they may increase the risk of postoperative pancreatitis, especially due to potential bile duct injuries during surgery. Additionally, anatomical variations, such as a low‐lying cystic duct or reduced bile duct diameter, and a history of pancreatitis or cholangitis can further heighten the risk. Post‐cholecystectomy Endoclip migration is also recognized as a contributing factor to post‐LC acute pancreatitis [17]. Very few cases have been documented in the literature in this contention. In this case report, a 23‐year‐old female patient with no significant medical history was admitted to the emergency department with complaints raising the suspicion of iatrogenic AP secondary to a recent laparoscopic cholecystectomy, which had been performed due to chronic cholecystitis associated with cholelithiasis.

Early identification of the cause of acute pancreatitis (AP) is important, as some cases require targeted treatment to prevent recurrence. Since gallstones are a leading cause, an abdominal ultrasound should be done within 48 h. Elevated liver enzymes support this diagnosis. If the first ultrasound is inconclusive but liver enzymes are high, a second scan may improve detection by about 20% [18]. The strategic use of imaging in acute pancreatitis is pivotal [19], with distinct roles for ultrasonography and computed tomography (CT) based on the clinical question at hand. In this case, an abdominal ultrasound was the appropriate initial imaging modality, performed in alignment with established guidelines recommending its use to investigate etiology, particularly for retained gallstones or biliary sludge. The findings of a non‐dilated common bile duct helped rule out a persistent obstruction as the cause of pancreatitis. Conversely, contrast‐enhanced CT is typically reserved for assessing the severity [19] of the disease or when complications such as necrosis are suspected, particularly in patients who fail to improve. Given that the diagnosis was unequivocally established by characteristic symptoms and markedly elevated serum amylase and lipase levels, and the pancreatitis was stratified as mild based on a low‐risk Ranson score of 2, a CT scan was not clinically indicated. This decision was further vindicated by the patient's rapid clinical and biochemical stabilization within 48 h, which negated the need for anatomical assessment for complications. Therefore, forgoing a CT scan represented a deliberate clinical judgment, aligning with principles of evidence‐based practice that advocate for avoiding unnecessary radiation exposure and resources in cases of uncomplicated, mild acute pancreatitis.

Although limited, this study evaluated the effectiveness of various biomarkers in predicting the severity of acute pancreatitis (AP). While previous research suggested serum trypsinogen‐2 levels may be useful, this study found that serum trypsin and TAP were less accurate than the APACHE II score. Despite a large patient cohort and robust data collection, limitations included a short observation window and a lower number of severe cases. Unlike earlier studies that found higher trypsin and TAP levels in severe AP, this study observed the opposite. Differences in sampling timing and patient etiology (e.g., alcohol‐induced AP) may explain the discrepancy. Given the inconsistent findings and rapid clearance of TAP from the bloodstream, routine use of trypsinogen or TAP as severity markers is not currently recommended. Instead, APACHE II remains the more reliable predictor. Further large‐scale, prospective studies are needed to clarify the role of these biomarkers in clinical practice [20]. In cases where biliary etiology is still suspected, more advanced diagnostic procedures such as MRCP or EUS should be employed.

To assess the severity of pancreatitis, we utilize Ranson scoring, a system that predicts the severity and mortality of acute pancreatitis through 11 parameters evaluated at admission and 48 h later [21]. In this case, subsequent laboratory investigations revealed a notable elevation in serum amylase and lipase levels, with the total leukocyte count (TLC) recorded at 16400/μl. The patient received a Ranson score of 2 at admission, putting her in a low‐risk category. Significantly, the patient's condition stabilized within 48 h with a Ranson score of 0.

Another diagnostic criterion widely used nationally and internationally is two out of three criteria comprising epigastric pain radiating towards the back, elevated levels of serum lipase and amylase (three times above normal), and pancreatic and radiographic evidence showing pancreatic parenchymal inflammatory signs [22]. Following diagnosis, the management protocol includes fluid resuscitation (FR), pain control, and nutritional support. The patients should initially remain NPO [23] with a nasogastric tube and receive antispasmodics, painkillers, and anti‐emetics like diphenhydramine HCL or ondansetron, with opioids being particularly effective. Epidural analgesia is also a good option for pain management and is also associated with reduced mortality [24]. However, the doctrine governing nutritional support in acute pancreatitis has undergone a profound evolution, displacing the traditional dogma of prolonged pancreatic rest with an evidence‐driven imperative for early enteral feeding. This paradigm shift is predicated on a sophisticated understanding of pathophysiology; early refeeding is now recognized as a critical intervention for preserving gut mucosal barrier integrity [19]. By maintaining intestinal function, it attenuates gut permeability and mitigates the risk of bacterial translocation, a key mechanism precipitating infectious complications such as infected pancreatic necrosis. This strategy is unequivocally endorsed by contemporary international guidelines, including those from the American College of Gastroenterology (ACG), which recommend initiating an oral diet in mild acute pancreatitis as soon as symptoms are tolerated, irrespective of the normalization of pancreatic enzyme levels. Therefore, the management strategy in this case—instituting a brief, initial NPO period for acute symptomatic control followed by a prompt reintroduction of oral nutrition upon clinical improvement—is fully consonant with modern, evidence‐based best practices. Fluid resuscitation is a cornerstone in the management of acute pancreatitis. A paradigm shift in clinical practice has occurred, with current evidence strongly favoring the use of Ringer's lactate over normal saline for intravenous hydration. Comparative clinical data indicate that the administration of Ringer's lactate is associated with superior outcomes, including a significant reduction in the length of hospital stay, lower rates of intensive care unit (ICU) admission, and a decreased incidence of local pancreatic complications when compared to resuscitation with normal saline [25]. Furthermore, the severity of acute pancreatitis is closely correlated with inflammatory markers such as C‐reactive protein (CRP) and the development of systemic inflammatory response syndrome (SIRS). Ringer's lactate has demonstrated an ability to more effectively attenuate this inflammatory cascade. Studies show that its administration leads to a greater reduction in CRP levels and a lower incidence of SIRS within the first 24 h of treatment. This beneficial anti‐inflammatory effect is largely attributed to its more physiologic, pH‐balanced composition, which helps mitigate the systemic inflammatory response characteristic of acute pancreatitis [26]. Current guidelines advise against prophylactic antibiotics in predicted severe AP or sterile necrosis due to the risk of multidrug‐resistant bacteria and fungal super infection [27]. However, in severe cases of AP with no progression towards betterment, antibiotic regimens are also started [19].

The therapeutic strategy for acute pancreatitis is contingent upon disease severity and the potential for ensuing complications. In instances of mild, uncomplicated pancreatitis, management is principally conservative, encompassing supportive measures such as aggressive fluid resuscitation, anti‐emetic therapy, and symptomatic control with antispasmodic agents.

For severe or necrotizing disease, the role of prophylactic antibiotics has become a central focus of clinical investigation. Current evidence increasingly supports the administration of carbapenems, a preference driven by their superior pancreatic tissue penetration and ability to achieve high therapeutic concentrations. The efficacy of this approach has been affirmed in a pivotal systematic review and meta‐analysis, which concluded that the prophylactic use of carbapenems significantly improves clinical outcomes in acute pancreatitis [28].

Notwithstanding the prioritization of carbapenems, the therapeutic utility of conventional antibiotics, particularly for managing associated intra‐abdominal infections, should not be overlooked. The value of cephalosporins, for example, has been demonstrated in relevant preclinical models. An illustrative experimental study investigating the role of cefixime in acute pancreatitis confirmed its potential efficacy, underscoring the continued relevance of this antibiotic class in specific clinical contexts [29]; such as this instance, following a Ranson score of 2, the patient was initially placed on NPO and started on Toradol and Provas for pain management. Intravenous Meropenem 1 g was administered three times daily for antibiotic coverage. And—rather than the currently recommended Ringer's lactate—solution fluid resuscitation was provided with 5% Dextrose saline, which is a limitation in the management of this case. As supported by recent American College of Gastroenterology guidelines and multiple meta‐analyses, Ringer's lactate is associated with a more favorable anti‐inflammatory response and better clinical outcomes in acute pancreatitis. The choice of fluid in this instance reflected local protocols, but this case underscores the importance of integrating updated evidence into institutional standards to optimize patient care.

## 6 Conclusion

Iatrogenic pancreatitis is a rare but potentially life‐threatening complication of laparoscopic cholecystectomy. This case report highlights the importance of prompt recognition and aggressive management of this complication, even in young and otherwise healthy individuals. A high index of suspicion, timely imaging, and multidisciplinary management are crucial in preventing severe consequences. Surgeons and healthcare providers must be aware of this rare complication and take measures to prevent it, such as meticulous surgical technique and careful patient selection. Early intervention and management can significantly improve patient outcomes and reduce morbidity associated with iatrogenic pancreatitis.

## Author Contributions

**Fazeela Bibi:** conceptualization. **Janmejay Kumar Singh:** project administration. **Aiman Sadiq:** visualization. **Qurat‐Ul‐Ain Akram:** supervision. **Amina Asad:** writing – original draft. **Babar fayaz:** investigation. **Bilal Aslam:** writing – review and editing. **Vohra Maham Hassan:** writing – review and editing. **Umama Alam:** data curation, investigation. **Muhammad Abdullah Ali:** validation. **Said Hamid Sadat:** writing – original draft. **Khalil Elabdi:** writing – original draft, writing – review and editing.

## Ethics Statement

The authors have nothing to report.

## Consent

Written consent was taken from the patient. The patient is in contact with the authors.

## Conflicts of Interest

The authors declare no conflicts of interest.

## Data Availability

The data was taken from a patient who presented to our hospital, all data and references are publicly available on databases such as Pub‐med and Google Scholar.
